# Mechanisms of Inflammation in Proliferative Vitreoretinopathy: From Bench to Bedside

**DOI:** 10.1155/2012/815937

**Published:** 2012-09-25

**Authors:** Stavros N. Moysidis, Aristomenis Thanos, Demetrios G. Vavvas

**Affiliations:** Retina Service, Massachusetts Eye and Ear Infirmary, Department of Ophthalmology, Harvard Medical School, 243 Charles Street, Boston, MA 02114, USA

## Abstract

Proliferative vitreoretinopathy (PVR) is a vision-threatening disease and a common complication of surgery to correct rhegmatogenous retinal detachment (RRD). Several models of the pathogenesis of this disease have been described with some of these models focusing on the role of inflammatory cells and other models focusing on the role of growth factors and cytokines in the vitreous which come into contact with intraretinal and retinal pigment epithelial cells. New experiments have shed light on the pathogenesis of PVR and offer promising avenues for clinical intervention before PVR develops. One such target is the indirect pathway of activation of platelet-derived growth factor receptor alpha (PDGR**α**), which plays an important role in PVR. Clinical trials assessing the efficacy of 5-fluorouracil (5-FU) and low-molecular-weight heparin (LMWH), daunorubicin, and 13-cis-retinoic acid, among other therapies, have yielded mixed results. Here we review inflammatory and other mechanisms involved in the pathogenesis of PVR, we highlight important clinical trials, and we discuss how findings at the bench have the potential to be translated to the bedside.

## 1. Introduction

Proliferative vitreoretinopathy (PVR) is a vision-threatening disease that can occur secondary to retinal detachment (RD). RD allows macrophages, retinal pigment epithelial (RPE) cells, glial cells, and fibroblasts to migrate to the vitreous, where they proliferate, survive, form extracellular matrix proteins and assemble into a membrane [1]. This membrane can attach to the retina and subsequently contract, which can cause a new retinal detachment or failure of a surgically corrected detachment [2]. PVR occurs most commonly as a complication of surgery to correct rhegmatogenous retinal detachment (RRD) and is the most common reason for the failure of this operation [3, 4]. In one study of 119 patients with RRD and no previous vitreoretinal surgery, there was a 52.9% prevalence of PVR and 26.9% prevalence of severe PVR with mean retinal detachment duration of 58.4 ± 129.1 days [5]. Visual outcomes and the anatomical success of surgery are worse for RD that is complicated by PVR and may require twice as many resources to care for as those cases of RD without PVR [6]. Here we review inflammatory and other mechanisms involved in the pathogenesis of PVR, we highlight important clinical trials, and we discuss how findings at the bench have the potential to be translated to the bedside.

## 2. The Macrophage Hypothesis for Development of PVR

Some of the hypotheses regarding the pathogenesis of PVR have focused on the role of macrophages [7–9]. In one experiment, rabbits were injected intravitreally with cells obtained from their peritoneal cavity, consisting of 85% macrophages, 10% lymphocytes, a few neutrophils, and less than 1% erythrocytes [7]. One week after injection, intravitreal strands had developed containing macrophages and fibroblasts, with massive epiretinal membranes developing between 4 to 9 weeks after injection in 17 of 24 eyes, posterior vitreous separation in 16 of 24 eyes, and retinal detachment in 15 of 24 eyes. The researchers suggested that macrophage-derived enzymes produced changes in the structure of the vitreous by proteolysis of matrix proteins and also that the development of fibrotic membranes was due to the synthesis of fibroblast growth factor by the macrophages, but not due to cellular transdifferentiation of macrophages into fibroblast-like cells [7]. Immunohistochemical analysis of surgical specimens of patients with post-traumatic PVR indicated the presence of macrophages and transferrin in periretinal membranes [8]. It was suggested that the secretion of PDGF by macrophages was central to the pathophysiology of PVR in these specimens, since PDGF increases the density of the cell surface receptor for transferrin [8, 10]. This hypothesis is also supported by the development of PVR-models in rabbits and rats in which injected macrophages acquire fibroblastic characteristics and contribute to the formation of fibrocellular membranes [9, 11]. Macrophages (CD68-positive) were intravitreally injected into rats' eyes and by day 7, the majority of the rats (29/32) had white proliferative membranes attached to their retina [11]. This was followed by the development of neoformative membranes by day 14, but the rats did not develop complete retinal detachment; 20 control rats that received PBS injection did not have any proliferation or membrane formation. Furthermore, by day 28 a dense fibrous connective tissue had formed that on histology had a multilayer of fibroblast-like cells which on immunohistochemical analysis stained positive for vimentin (marker for mesenchymal cells), but not cytokeratin (marker of epithelial cells) or CD68 (marker of macrophages), suggesting the primary cells of the PVR membranes were fibroblasts [11]. Injected macrophages retained a round shape and CD68 on day 3, but on day 28 had developed a spindle shape with staining of vimentin and absence of CD68; the macrophages had acquired a fibroblast-like phenotype and contributed to the fibrocellular membranes directly [11]. It is likely that the role of macrophages in the pathogenesis of PVR is multifactorial and involves a combination of macrophage-secreted factors including enzymes and growth factors (e.g., PDGF) and also transdifferentiation of macrophages into fibroblast-like cells.

## 3. Injection of Cells into the Vitreous as a Model of PVR

In early models of PVR, a piece of dermal tissue was delivered to the vitreous of rabbit eyes through a small cauterized hole in the pars plana; growth of this tissue ensued, with the development of vitreous strands between the tissue and the retina, and ultimately retinal detachment in the majority of cases [12]. This was followed by experiments in which fibroblasts were intravitreally injected into rabbits [13–15]. Autotransplanted, cultured skin fibroblasts injected intravitreally resulted in vitreous strands, preretinal pucker, and traction detachment in 32 of 51 eyes [13]. In another rabbit model, gas compression was used to simulate vitrectomy and followed a week later by injection of autologous tissue-cultured fibroblasts; by post-op day 28, 10 of 10 eyes injected with 50,000 fibroblasts had developed transvitreal strands and severe retinal detachment [14]. Meanwhile, in eyes injected with 25,000 fibroblasts, 7 of 11 showed transvitreal strands, and 10 of 11 developed retinal detachment [14]. In the epiretinal membranes of patients undergoing vitreoretinal surgery for retinal detachment complicated by PVR, all 16 samples contained myofibroblasts expressing the contractile protein *α*-smooth muscle actin [16]. *In vitro*, the addition of bovine vitreous to cultures of RPE cells and fibroblasts stimulated the proliferation of these two cell types [17]. Furthermore, pathologic vitreous from patients with PVR stimulated contraction of cultured fibroblasts *in vitro *[18]. In rats, intravitreal injection of rat RPE (RPE-J) cells and platelet-rich plasma resulted in proliferative membranes and retinal detachment by post-injection day 28 [19]. Immunohistochemical analysis of membranes at days 14 and 28 revealed RPE cells expressing cytokeratin-18, glial cells expressing GFAP, fibroblasts expressing vimentin, and ED-1 positive macrophages [19]. This evidence, along with the macrophage model of PVR, suggests that it may be the introduction of cells into the vitreous that triggers processes leading to PVR, rather than the particular cell injected.

## 4. The Growth Factor and Cytokine Hypothesis for Development of PVR

**Figure 1:**
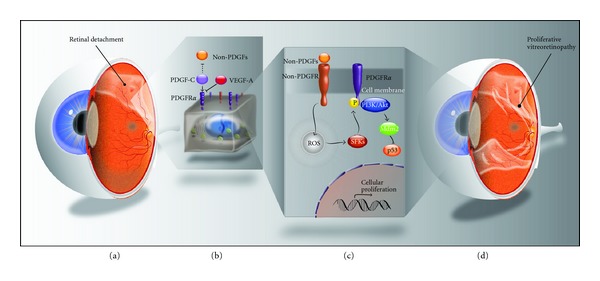
Indirect activation of PDGFR*α* by non-PDGFs triggers the events leading to proliferative vitreoretinopathy (PVR). A retinal tear or detachment (a) creates an opening via which vitreal growth factors and cytokines interact with intraretinal cells and retinal pigment epithelial (RPE) cells. Vitreal VEGF-A competitively inhibits the binding of platelet-derived growth factors (PDGFs), including the predominant isoform isolated in the vitreous of patients with PVR, PDGF-C, to the receptor PDGFR-*α* (b). In doing so, VEGF-A prevents direct activation of PDGFR*α* by PDGFs. Direct activation of PDGFR*α* promotes rapid clearance of this receptor from the cell surface and subsequent intracellular degradation; this rapid receptor cycling interferes (b) with the ability of non-PDGFs to activate the PDGFR*α* through an indirect pathway as follows. Non-PDGFs, including basic fibroblast growth factor (bFGF), epidermal growth factor (EGF), insulin, and hepatocyte growth factor (HGF), activate their receptors, which results in an elevation of the level of intracellular reactive oxygen species (ROS), which leads to activation of Src family kinases (SFKs) that promote phosphorylation and activation of PDGFR*α* (c). This pathway of indirect activation results in persistent PDGFR*α* signaling and induces prolonged activation of phosphatidylinositol 3-kinase (PI3K)/Akt, which phosphorylates murine double minute (Mdm2), which then suppresses p53 levels (c). This promotes an environment of cell survival, proliferation, organization into a membrane, and subsequent membrane contraction, the processes intrinsic to PVR (d). Therefore, VEGF-A inhibits physiological, direct activation of PDGFR*α* by PDGFs and favors pathological, persistent, indirect activation of the receptor by non-PDGFs, triggering the events leading to PVR.

In the proposed growth factor and cytokine model for the development of PVR, a break in the retina, such as that occurring in RRD, creates an opening for vitreous to come into contact with intraretinal cells and retinal pigment epithelial (RPE) cells. Vitreal growth factors and cytokines, now with access to these cells, promote an environment of cell migration, proliferation, survival, and formation of extracellular matrix proteins (Figure 1) [20]. As these structures form, they may physically attach to the retina, contract, and cause retinal tears. Support for this hypothesis stems from the presence of many growth factors and cytokines in the pathological vitreous or epiretinal membrane, including platelet-derived growth factor (PDGF) isoforms [21, 22], hepatocyte growth factor (HGF) [22, 23], vascular endothelial growth factor (VEGF) [24], epidermal growth factor (EGF) [25], pigment epithelium-derived factor (PEDF) [26], transforming growth factor *β* (TGF*β*) [27, 28], tumor necrosis factor *α* (TNF*α*) [29, 30], TNF*β* [29], granulocyte colony-stimulating factor (G-CSF) [29], fibroblast growth factors (FGF) [29, 31], basic fibroblast growth factor (bFGF) [32], insulin [25], insulin-like growth factor-1 (IGF-1) [33], connective tissue growth factor (CTGF) [22, 23], glutamine synthetase [32], interleukin 1 (IL-1) [34], IL-6 [29, 31], IL-8 [29, 35], IL-10 [29], interferon *γ* (IFN*γ*) [28, 29], monocyte chemotactic protein [35, 36], macrophage-colony stimulating factor [35], granulocyte colony-stimulating factor (G-CSF) [29], chemokine ligand 2 (CCL2) [29], CCL3 [29], CCL4 [29], CCL5 [29], and protein [31].

## 5. Tumor Necrosis Factor Alpha as a Promoter of PVR

Tumor necrosis factor (TNF)*α* is a cytokine that promotes inflammation, in part, by activating endothelial cells to display leukocyte adhesion molecules such as E-selectin, intercellular adhesion molecule-1, and vascular cell adhesion molecule-1 [37, 38]. TNF*α* was found in 22 of 26 epiretinal membranes of patients with proliferative vitreoretinopathy, with positive TNF*α* staining both intracellularly and in the extracellular matrix [39]. TNF*α* is associated with the production and secretion of the receptors sTNF-RI and sTNF-RII, which are found on the majority of nucleated cells; after activation by TNF*α*, these receptors are cleaved by metalloproteinases [40] and found in the soluble form in serum [41]. sTNF-RI and sTNF-RII are thought to neutralize the inflammatory effects of TNF*αin vitro* and *in vivo* and can be used clinically as markers of disease activity [42, 43]. The levels of sTNF-RI and sTNF-RII were significantly higher (*P* < 0.0003) in the vitreous of patients with PVR (244–4290 and 128–4429 pg/mL, resp.) compared to cadaveric controls (101–836 and 96–551 pg/mL, resp.) [44]. Groups in the aforementioned study were not matched for age; another study suggests that sTNF-RI and sTNF-RII are significantly increased in the serum of healthy older people (mean 71 years) and centenarians compared to younger, healthy controls (mean 27.9 years) [45]. Genetic analysis of blood samples from 138 patients with post-rhegmatogenous retinal detachment PVR demonstrated a significant association (*P* = 0.0283) with the nonsynonymous, single nucleotide polymorphism (SNP) rs2229094(T→C) compared to controls [46]. This is a SNP in the lymphotoxin alpha gene at the tumor necrosis factor locus (6p21.3), which encodes a cysteine to arginine change—from a neutral, hydrophobic amino acid to a hydrophilic, positively charged amino acid—and may have an effect on protein topology or its interactions [46]. Future studies on protein function may further elucidate the role of this SNP at the TNF*α* locus in PVR.

## 6. PDGFRs Are Involved in the Pathogenesis of PVR

PDGF is an important link in the cell-cell interactions of retinal cells and functions as a trophic factor during the development of the retina [47, 48]. PDGFR has been identified on the cell membranes of RPE cells, retinal glial cells, and fibroblasts, some of the cell types involved in PVR [49, 50]. PDGF and activated PDGFR have been noted in the epiretinal membranes, RPE, and glial cells of patients with PVR, with high levels of PDGF in the vitreous closely associated with PVR (8/9 patients with PVR had detectable levels of vitreal PDGF compared to 1/16 patients with a different retinal disease requiring surgery/vitrectomy) [51, 52]. Only the PDGF-C isoform was isolated, which is produced mainly by the protease plasmin [53]. This finding was corroborated by a high level of PDGF-C in the vitreous of rabbit models of PVR induced by fibroblast injection [52, 54]. Additionally, in experimental models, cells that lacked the PDGFR gene had a low potential for PVR and reexpressing the wild type PDGFR in these cells greatly increased the potential for PVR [21, 50]. Inhibition of PDGFRs decreased cellular PVR potential [55, 56]. Of the three different PDGFRs: cells expressing PDGFR*α* induce PVR much more effectively than cells expressing PDGFR*β* in rabbits, and cells expressing the heterodimer PDGFR*αβ* had intermediate potency in inducing PVR [21]. This is supported clinically by analysis of human specimens demonstrating that a greater percentage of PDGFR*α* is activated [51]. In addition, PDGF-C, the predominant PDGF isoform isolated in the vitreous of patients with PVR, activates PDGFR*α* and PDGFR*αβ* but not PDGFR*β* [52, 57].

## 7. Indirect Activation of PDGFR by Non-PDGFs Triggers the Events Leading to Experimental PVR

Non-PDGFs can also activate PDGFR*α*; for example, bFGF, EGF, insulin, and HGF induce tyrosine phosphorylation of PDGFR*α* [25]. Non-PDGFs activated both full-length PDGFR*α* and mutant receptors that lacked the extracellular domain to a comparable extent, through the following pathway: non-PDGFs activate their receptors, resulting in an increase of intracellular reactive oxygen species (ROS), then activation of Src family kinases (SFK), which leads to phosphorylation of PDGFR*α* (Figure 1) [58]. New evidence suggests that this indirect pathway involving non-PDGFs as agonists of PDGFR*α* is the primary pathway for activation of this receptor and an important part of the pathogenesis of PVR. VEGF-A prevents binding of PDGF to PDGFR*α*, inhibiting the direct pathway of PDGFR*α* activation and downstream extracellular signal-related kinase (Erk) activation [59]. Neutralizing VEGF-A by adding anti-VEGF-A antibodies to the vitreous of rabbits with PVR resulted in a significant increase in the activation of PDGFR*α*; VEGF-A influences the mechanism of PDGFR*α* activation, inhibiting the direct pathway and creating an environment favoring non-PDGFs to indirectly activate PDGFR*α* [59]. While direct activation of PDGFR*α* results in rapid clearance of the receptor from the surface and subsequent degradation, indirect activation by non-PDGFs promotes persistent receptor signaling and induces prolonged activation of phosphatidylinositol 3-kinase (PI3K)/Akt, which activates murine double minute (Mdm2) to suppress p53 levels, driving processes intrinsic to PVR-survival, proliferation, and contraction (Figure 1) [59, 60]. 

## 8. Therapeutic Targeting of the PDGF/PDGFR Pathway

Attempts to prevent retinal detachment and PVR with antibodies directed against PDGFs have yielded mixed results. In photoreceptors of transgenic mice overexpressing PDGF-B, the universal ligand for all three PDGF receptors, intravitreal injection of an aptamer against PDGF-B was protective against retinal detachment [61]. In a rabbit model, antibodies against vitreal PDGFs inhibited them effectively but did not prevent PVR compared to controls (Table 1), suggesting that the PDGFRs in this model were activated by non-PDGFs [25]. Attempts were then made to inhibit the indirect pathway of PDGFR activation, a pathogenesis that involves an increase in ROS. In a comparison of cells null for all PDGFRs and cells containing a truncated PDGFR*α* that could only undergo indirect activation, both bFGF (which increases ROS) and then separately rabbit vitreous, caused the cells with truncated PDGFR*α* to robustly contract but did not cause contraction in control cells null for the receptor. The experiment was then repeated in the presence of N-acetyl-cysteine (NAC), an antioxidant that inhibits ROS formation. At concentrations of 2.5 mmol/L NAC and above (NAC-induced toxicity began to occur at 20 mmol/L), contraction of the PDGFR*α*-cell lines was prevented, as was the proliferative advantage of PDGFR*α*-containing cells over control cells [62]. These findings were then applied *in vivo* to PVR-model rabbits, where a vitreal concentration of 10 mmol/L of NAC was found to significantly reduce the PVR response compared to injection of buffer, with suppression persisting 3 weeks post-NAC injection; while the development of membranes occurred in most of the treated rabbits, they did not progress to retinal detachment, and analysis of PVR membranes revealed that control rabbits had 2.6 times the PDGFR*α* activation compared to treated rabbits (Table 1) [62]. NAC also prevented contraction of primary RPE cells isolated from a human PVR membrane which was subjected to the donor vitreous of five patients with PVR; NAC may be used to suppress receptor activation and retinal detachment but not to target pathological cells' viability [62]. 

## 9. Neutralizing a Subset of Non-PDGFs and Cytokines to Prevent PVR

Approaches with a cocktail of neutralizing reagents to target multiple growth factors and cytokines have also been studied. One *in vitro* study assessed for the minimum possible neutralizing set of antibodies that could be delivered to prevent cellular contraction in the presence of pathologic PVR vitreous. The minimum neutralizing set blocking PVR-related signaling was found to be a cocktail of antibodies that neutralized PDGFs, TGF*α*, EGF, HGF, FGF-2, TGF*β*, IL-8, and IGF-1 [63]. The rationale for neutralizing PDGFs, despite evidence suggesting that the direct pathway of PDGFR*α* activation plays only a minor role in PVR, was to preempt against the possibility that inhibiting the indirect pathway would then potentiate and increase the bioactivity of the direct pathway. These findings were then applied *in vivo* to rabbit PVR-models by treating twelve rabbits with the minimum neutralizing cocktail and another twelve with nonimmune IgG. Of the control rabbits, 8 (67%) developed stage 3 PVR or higher with retinal detachments and the other 4 (33%) developed stage 2 PVR. In contrast, none of the treated rabbits developed retinal detachment, with 3 (25%) having no pathology, 5 (42%) developing an epiretinal membrane, and 4 (33%) developing stage 2 PVR (Table 1) [63]. Furthermore, treated eyes did not develop vitreal or anterior chamber white cells, and the histology of one of these treated rabbits revealed no retinal damage compared to histology of the noninjected eye of the same rabbit [63]. 

## 10. 5-Fluorouracil (5-FU) and Low-Molecular-Weight Heparin (LMWH): Clinical Trials

5-Fluorouracil (5-FU) is an antimetabolite that inhibits DNA synthesis and fibroblast proliferation [64]. Low-molecular-weight heparin (LMWH) is an anticoagulant that binds fibronectin, bFGF, PDGF, and other growth factors [65]. Animal studies have found some efficacy of 5-FU for the treatment of vitreoretinal scarring [66, 67], but results in human clinical trials have been mixed (Table 2) [68–70]. In one prospective, randomized, double-masked, placebo-controlled trial, 174 high-risk patients were randomized to receive 5-FU and LMWH (*n* = 87) versus placebo (*n* = 87) after primary vitrectomy for rhegmatogenous retinal detachment [68]. The study reports a significantly (*P* = 0.02) lower incidence of postoperative PVR in the 5-FU and LMWH therapy group (11/87, 12.6%) compared to placebo (23/87, 26.4%). In the treatment group, 19.5% (17/87) of patients required more than one operation with 52.9% (9/17) due to PVR compared to 25.3% (22/87) of patients in the placebo group requiring reoperation with 72.7% (16/22) due to PVR. There was no significant difference in visual acuity (VA) outcomes in the two groups although patients with postoperative PVR had worse VA, nor were there significant differences in the complication rates of the two groups [68]. In another randomized, controlled trial of 5-FU and LMWH in patients with established anterior or posterior grade C PVR, patients were randomized to receive a perioperative infusion with or without 200 *μ*g/mL of 5-FU and 5 IU/mL LMWH during vitreoretinal surgery and silicone oil exchange [69]. The trial looked at the primary outcome of posterior retinal reattachment after removal of silicone oil without any reoperations at 6 months and found no significant difference (*χ* ^2^ = 2.9, *P* = 0.59) between the treatment group (56%, *n* = 73) and the placebo group (51%, *n* = 84) [69]. These trials were followed by a large, randomized, controlled trial of 5-FU and LMWH versus placebo in 615 patients presenting with unselected primary rhegmatogenous retinal detachment [70]. The main outcome measure was retinal reattachment after primary vitrectomy without any reoperations at 6 months with secondary outcome measures including occurrence and grade of PVR and best-corrected visual acuity. Retinal reattachment after primary vitrectomy was 82.3% in the combined 5-FU and LMWH group (*n* = 327) and 86.8% in the placebo group (*n* = 288; *P* = 0.12), with no statistically significant difference in development of PVR (7% in treatment group compared to 4.9% in placebo group; *P* = 0.072), nor was there a significant difference in the median final visual acuity of the two groups [70]. Evidence for adjuvant therapy with 5-FU and LMWH for the prevention of PVR is mixed; additional trials targeting prevention in patients with high risk for PVR may provide greater insight [71]. 

## 11. Daunorubicin in the Treatment of PVR: *In Vitro*, *In Vivo*, and Clinical Trials

Daunorubicin or daunomycin is an anthracycline that inhibits cell proliferation and migration [72, 73]. Early use of intraocular daunorubicin *in vitro* and *in vivo* in rabbits determined that the concentration that caused a 50% inhibition of colony-forming units was 700 nM; the half-life of daunomycin was determined to be 131 minutes in the vitreous, conveying that critical concentrations of the drug can be maintained for more than 4 hours after injection, with safe elimination across the retina [73]. Daunorubicin used in humans to reduce the failure rate of surgery for traumatic proliferative vitreoretinopathy due to postoperative cellular proliferation reported anatomic success in 14 out of 15 patients; daunorubicin was delivered at 7.5 *μ*g/mL over a ten-minute period after vitrectomy and before silicone oil or gas injection, with no reported toxicity to the optic nerve, retina, lens, or cornea [74]. In a controlled clinical trial, 286 patients with advanced preoperative PVR were randomized into standardized surgery with adjunctive daunorubicin or surgery alone (Table 2) [75]. Outcomes included retinal attachment with no additional vitreoretinal surgery to 6 months postop, number of and time to additional surgery within 1 year of the first operation, and best-corrected visual acuity at 1 year postop. The trial demonstrated no significant difference (*P* = 0.07) in retinal attachment at 6 months post-op between the two groups with the daunorubicin group having 62.7% (89/142) attachment and 54.1% (73/135) in the control group. In secondary outcomes, there was a statistically significant difference in the need for another vitreoretinal operation within 1 year of the first surgery (*P* = 0.005), with the daunorubicin group requiring fewer such operations 34.5% (50/145) compared to the control group 46.1% (65/141); there was no difference in best-corrected visual acuity [75]. There are a limited number of trials studying the efficacy of daunorubicin in prevention of PVR, but it appears to be ineffective when used as a single agent.

## 12. Corticosteroids, 13-Cis-Retinoic Acid, Cyclin-Dependent Kinases, and Novel Compounds

Experiments in rabbits found that a single intravitreal injection of 1 mg of triamcinolone acetonide effectively inhibited fibroblast growth in a fibroblast autotransplantation model, reducing retinal detachment from 83.7% (36/43) to 34.1% (15/44) as well as the rate of retinal neovascularization from 72.1% (31/43) in controls to 18.2% (8/44) in treated rabbits [76]. In a prospective clinical trial, however, a much weaker response was seen; patients treated with systemic steroids had a 63.3% incidence of retinal fibrosis compared to 75.4% of patients given placebo following retinal detachment surgery [77]. 13-Cis-retinoic-acid (13cRA) has been found to inhibit proliferation of RPE cells *in vitro *[78, 79]. A randomized, controlled, clinical trial of 35 patients with primary retinal detachment and PVR undergoing similar surgery, assigned 16 patients to receive 10 mg of oral 13cRA twice daily for eight weeks postoperatively and the other 19 patients to the control group; the primary outcome measure was retinal attachment at one-year followup (Table 2) [80]. At one-year followup, there was a statistically significant difference in retinal attachment (*P* = 0.047) between the two groups, with 93.8% (15/16) of eyes in the 13cRA group maintaining retinal attachment compared to 63.2% (12/19) of eyes in the control group [80]. Other agents, including the cyclin-dependent kinase inhibitor roscovitine and a novel anti-angiogenic compound IMS2186, have shown promise in animal models for inhibiting the proliferation of retinal pigment epithelial cells and fibroblasts, respectively [81, 82].

## 13. Conclusions

Basic science and clinical studies continue to provide growing insight into the pathophysiology of proliferative vitreoretinopathy. In posttraumatic PVR, macrophages can secrete growth factors (e.g., PDGF) and can transdifferentiate into fibroblast-like cells, thereby contributing to vitreoretinal membrane formation. In animal models, the injection of cells into the vitreous, whether they are macrophages, dermal tissue, fibroblasts, or RPE-J cells, results in pathology that mimics PVR. Tumor necrosis factor alpha, a pro-inflammatory cytokine, has been identified in close association with the membranes of patients with PVR, and genetic analysis has identified a single nucleotide polymorphism at the tumor necrosis factor locus that alters protein structure. Inflammatory processes in the vitreous are accentuated by the presence of growth factors, including PDGFs, HGF, bFGF, and EGF, to name a few. These growth factors, and especially the non-PDGFs, appear to activate PDGFRs on the surface of RPE cells, retinal glial cells, and fibroblasts, leading to cell survival, proliferation, organization into a membrane, and subsequent membrane contraction. Vitreal VEGF-A appears to competitively inhibit the binding of PDGFs to PDGFR-*α*. This promotes activation of PDGFR-*α* by non-PDGFs through an indirect pathway that results in persistent PDGFR*α* signaling—a pathway that leads to prolonged suppression of p53 and triggers the events leading to PVR. One key difference between animal models of PVR and the disease as it occurs in humans is that in the majority of animal models, PVR is induced by injection of cultured fibroblasts. Meanwhile, in humans, PVR may follow retinal detachment or primary repair of rhegmatogenous retinal detachment; the inflammatory process in humans is more likely to involve cells local to the retina and vitreous rather than cells introduced from outside the eye. While clinical trials have thus far offered mixed results in attempting to prevent the pathogenesis of proliferative vitreoretinopathy, experiments at the bench have provided novel strategies *in vitro* and in animal models and offer new avenues clinically for future attempts to prevent this sight-threatening disease. Clinical strategies to prevent PVR will probably require a multimodal, combinatorial approach, such as ROS inhibition and blocking the direct and indirect pathway of PDGFR*α* activation. Furthermore, pars plana vitrectomy will remain a critical component of the treatment in rhegmatogenous retinal detachment and PVR since residual vitreous is a risk factor of PVR. Finally, attention should be given to optimizing the correct dosing and administration of drugs, since some of the past failures may be due to the manner and time of administration rather than due to lack of true efficacy of the drugs tested.

## Figures and Tables

**Table 1:** Outcomes of therapeutic agents used in animals to prevent proliferative vitreoretinopathy (PVR).

Agent(s)	Dose and target	*In vivo* model	Treatment groups	Outcomes	Ref.
ARC126, ARC127, ARC128	20 *μ*g, aptamers against PDGF-B	Rho/PDGFB transgenic mice treated on P7, sacrificed P12	Intravitreal inj. on P7: PBS OD; ARC126, ARC-127, ARC128 OS	PBS: mean area of GSA+ cells 1.82 mm^2^, 11/19 totRD, 4/19 pRD, 79% RD ARC126: mean area of GSA+ cells 0.79 mm^2^ (*P* = 0.0003*), 0 totRD, 1/6 pRD, 17% RD ARC127: mean area of GSA+ cells 0.55 mm^2^ (*P* = 0.0001*), 0/7 totRD and pRD, 0% RD ARC128: mean area of GSA+ cells 2.00 mm^2^ (*P* > 0.05), 3/6 totRD, 2/6 pRD, 83% RD	[61]

(a) Mouse mAb IgG1 HH1-57; (b) Trap PDGFR*α*-Fc5	(a) 200 *μ*g, inhibits PDGF-C (b) 386 *μ*g, inhibits all PDGF isoforms	Rabbits with OD PVR-induced by injection of PRP and fibroblasts	Intravitreal inj. on day 0: control (*n* = 8) versus (a) mAb IgG1 HH1-57 (*n* = 9) and (b) Trap (*n* = 13)	Control:* *1*Stage1, *2*S2, *4*S3, *1*S4 *(day 3);* *1*Stage2, *2*S3, *5*S4 * (d5);* *1*Stage2, *1*S3, *6*S4 * (d7) (a) HH1-57: 2*S1, *2*S2, *4*S3, *1*S4 *(d3, *P* = 0.229); 2*S2, *4*S3, *3*S4 *(d5, *P* = 0.108); 2*S2,*3*S3, *3*S4, *1*S5 *(d7, *P* = 0.406) (b) Trap: 1*S0, *6*S1, *4*S2, *1*S3, *1*S4 *(d3, *P* = 0.041*); 3*S1, *3*S2, *4*S3, *3*S4 *(d5, *P* = 0.063); 4*S1, *2*S2, *3*S3, *4*S4 *(d7, *P* = 0.058)	[25]

N-acetyl-cysteine (NAC)	10 mmol/L, reduces ROS levels	Rabbits with OD PVR-induced by injection of PRP and fibroblasts	Intravitreal inj. on days 0, 2, 4, and 7: control (*n* = 10) versus NAC (*n* = 10)	Control: 2*Stage0, *8*S1 *(d1); 1*S0, *7*S1, *2*S2 *(d3); 8*S1, *1*S2, *1*S4 *(d5); 5*S1, *2*S2, *2*S3, *1*S4 *(d7); *S1, *3*S2, *2*S3, *1*S4, *1*S5 *(d14); 4*S1, *2*S2, *1*S3, *1*S4, *2*S5 *(d21); 3*S1, *3*S2, *1*S4, *3*S5 *(d28); 2.6x ↑ phosphorylation of PDGFR in membranes of control compared to NAC NAC: 8*S0,*2*S1 *(d1, *P* = 0.0232*); 9*S0,*1*S1 *(d3, *P* = 0.0011*); 9*S0,*1*S1 *(d5 *P* = 0.0007*); 7*S0,*3*S1 *(d7, *P* = 0.0015*); 4*S0,*6*S1 *(d14, *P* < 0.0011*); 3*S0,*5*S1,*2*S2 *(d21, *P* < 0.032*); 3*S0,*4*S1,*3*S2 *(d28, *P* < 0.0185*); no progression to RD	[62]

Minimum neutralizing antibody set (MNAS)	Antibody set against PDGFs, TGF*α*, EGF, HGF, FGF-2, TGF*β*, IL-8, and IGF-1	Rabbits with OD PVR-induced by injection of PRP and fibroblasts	Intravitreal inj. on day 0: control (*n* = 12) versus MNAS (*n* = 12)	Control: 4/12 (33%) stage 2 PVR, 8/12 (67%) stage 3 PVR or higher with RD MNAS: 3/12 (25%) no pathology, 5/12 (42%) epiretinal membrane, 4/12 (33%) stage 2 PVR, no RD	[63]

PDGF: platelet-derived growth factor. ARC128: nonfunctional version of ARC127. P7: postnatal day 7. P12: postnatal day 12. inj.: injection. PBS: phosphate-buffered saline. OD: right eye. OS: left eye. GSA+ cells: ectopic cells in the inner retina staining positive for Griffonia simplicifolia lectin. totRD: total, pRD: partial, RD: retinal detachment. *statistically significant difference compared to control. mAb: monoclonal antibody. PDGFR: platelet-derived growth factor receptor. PRP: platelet-rich plasma. #*S0*–*#S5*: number of cases observed at a given Fastenberg Stage of PVR 0–5. d: day. ROS: reactive oxygen species. TGF*α*: transforming growth factor *α*. EGF: epidermal growth factor. HGF: hepatocyte growth factor. FGF-2: fibroblast growth factor 2. TGF*β*: transforming growth factor *β*. IL-8: interleukin 8. IGF-1: insulin-like growth factor-1. Ref: Reference number.

**Table 2:** Outcomes of randomized, controlled, clinical trials in humans with and without proliferative vitreoretinopathy (PVR).

Agent(s)	Dose and target	Patients	Treatment groups	Outcomes	Ref.
5-FU and LMWH	200 *μ*g/mL 5-FU and 5 IU/mL LMWH, 5-FU inhibits DNA synthesis and fibroblast proliferation; LMWH binds fibronectin, bFGF, PDGF, and other growth factors	174 patients at High risk for PVR	Intravitreal inf.: placebo versus 5-FU and LMWH	Placebo: 23/87 (26.4%) postoperative PVR, 22/87 (25.3%) reoperation with 16/22 (72.7%) due to PVR 5-FU and LMWH: 11/87 (12.6%, P = 0.02*) postoperative PVR, 17/87 (19.5%) reoperation with 9/17 (52.9%) due to PVR No difference in visual acuity outcomes, nor complication rates	[68]

5-FU and LMWH	200 *μ*g/mL 5-FU and 5 IU/mL LMWH, For target, see Row 1 above	157 patients with established grade C PVR	Intravitreal inf.: placebo versus 5-FU and LMWH	Placebo: 51% (n = 84) retinal reattachment (RRA) at 6 months with no reoperation 5-FU and LMWH: 56% (n = 73) RRA at 6 months with no reoperation (χ^2^ = 2.9, P = 0.59)	[69]

5-FU and LMWH	200 *μ*g/mL 5-FU and 5 IU/mL LMWH, For target, see Row 1 above	615 patients with unselected primary RRD	Intravitreal inf.: placebo versus 5-FU and LMWH	Placebo: 86.8% RRA, 4.9% development of PVR, (n = 288) 5-FU and LMWH: 82.3% RRA (P = 0.12), 7% development of PVR (P = 0.072), (n = 327) No difference in final mean visual acuity	[70]

Dauno-rubicin	7.5 *μ*g/mL, inhibits cell proliferation and migration	286 patients with advanced preoperative PVR after RRD	Surgery only versus surgery with intravitreal inf. of daunorubicin	Surgery only: 73/135 (54.1%) RRA with no reoperation at 6 months Surgery and daunorubicin: 89/142 (62.7%) RRA with no reoperation a 6-month post-op (P = 0.07) Daunorubicin: less reoperation 1st year postop (46.1% versus 34.5%, P = 0.005*)	[75]

13-cis-retinoic acid (13cRA)	10 mg orally, 2x daily, for 8 weeks, inhibits proliferation of RPE cells	35 patients with primary retinal detachment and PVR	Surgery only versus surgery and oral 13cRA	Surgery only: 12/19 (63.2%) RRA at one-year follow-up Surgery and 13cRA: 15/16 (93.8%) RRA at one-year followup (P = 0.047*)	[80]

5-FU: 5-fluorouracil. LMWH: low-molecular-weight heparin. bFGF: basic fibroblast growth factor. PDGF: platelet-derived growth factor. inf.: infusion. RRD: rhegmatogenous retinal detachment. post-op: postoperative. *statistically significant difference compared to control. RRA: retinal reattachment. RPE: retinal pigment epithelium. 13cRA: 13-cis-retinoic acid. Ref: Reference number.
